# Retiform purpura in a patient with iododerma and atypical perinuclear antineutrophil cytoplasmic antibody

**DOI:** 10.1016/j.jdcr.2025.12.003

**Published:** 2025-12-11

**Authors:** Sasha R. Vigil, Le Wen Chiu, Jose L. Cortez, Shelly A. Stepenaskie, Nikifor K. Konstantinov

**Affiliations:** a Department of Dermatology, University of New Mexico, Albuquerque, New Mexico; b Tricore Reference Laboratories, University of New Mexico, Albuquerque, New Mexico; c Department of Internal Medicine, University of New Mexico, Albuquerque, New Mexico

**Keywords:** hydralazine, iododerma, medical dermatology, retiform purpura

## Introduction

Iododerma is a rare, rapidly progressive dermatosis that occurs after exposure to iodinated compounds such as iodinated contrast media, potassium iodide, amiodarone, topical povidone-iodine, and radioactive iodine 131.^1^ It develops more commonly in patients with impaired renal function, as iodine accumulates in the blood and acts as a hapten to induce a delayed hypersensitivity reaction.^2^ Iododerma presents variably with purpuric, acneiform, or vegetative plaques, as well as hemorrhagic bullae and necrotic lesions, often posing a diagnostic challenge.^1^^,^^2^

We present a patient with atypical perinuclear antineutrophil antibody (ANCA) on hydralazine therapy who developed iododerma with retiform purpura, mimicking an ANCA-associated vasculitis (AAV).

## Case report

A 44-year-old man with a history of hypertension on hydralazine and end-stage renal disease on hemodialysis was found unresponsive after a fall. Computed tomography (CT) angiography with iodinated contrast showed a subarachnoid hemorrhage, followed by clinical deterioration, necessitating admission to the intensive care unit. Two days following hospitalization, the patient developed a rapidly progressive purpuric rash.

**Fig 1:**
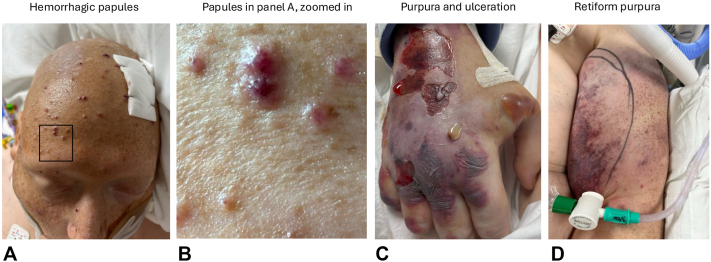
Clinical features of the patient. **A,** Hemorrhagic vesiculopapules scattered on nose, forehead, and scalp. **B,** Closer examination of hemorrhagic vesiculopapules from highlighted area on panel **(A)**. **C,** Retiform purpuric plaques with angulated ulceration and tense bullae on dorsal portion of the right hand. **D,** Retiform purpura on the upper portion of the left arm.

**Fig 2:**
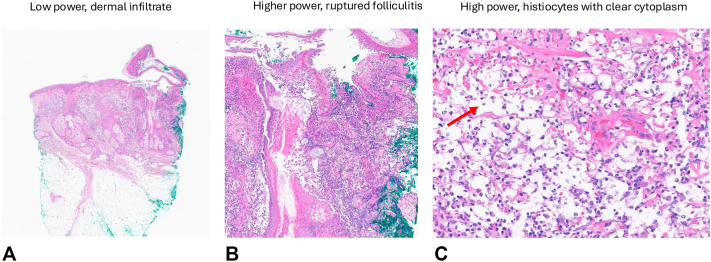
Histopathological features. **A,** Punch biopsy of the scalp showing a dermal infiltrate. **B,** Changes in acute ruptured folliculitis with dense neutrophilic inflammatory infiltrate. Fibrinoid necrosis of few small and medium vessels is seen in association with the ruptured folliculitis . **C,** Histiocytes with clear cytoplasm (*red arrow*), resembling haloed cryptococcus-like structures. Punch biopsies from the scalp and hand showed similar findings of haloed structures along with the neutrophils and vasculitic changes throughout the dermis and subcutis. (**A-C,** Hematoxylin-eosin stain; original magnifications: **A,** ×4; **B,** ×10; **C,** ×40.)

Examination revealed retiform purpuric plaques on the bilateral proximal arms, hemorrhagic bullae and ulceration on the right hand, and hemorrhagic vesiculopapules on the head (Fig 1, *A-D*). Punch biopsies of the scalp and hand contained dermal haloed cells resembling cryptococcal-like structures, which were negative for fungal organisms, with Gomori's Methenamine Silver and Periodic acid-Schiff staining also performed. Associated acute folliculitis with rupture and dermal neutrophils were also observed (Fig 2, *A-C*). Tissue culture biopsy for bacterial, fungal, and acid-fast bacilli was negative, and both serum and cerebrospinal fluid cryptococcal antigen tests were negative. Laboratory workup was notable for an atypical perinuclear ANCA (p-ANCA) pattern, with negative antimyeloperoxidase and antiproteinase-3 antibodies. A contrast-enhanced CT chest was obtained and revealed a 3.0 cm spiculated nodule within the left upper lobe, with scattered patchy ground glass opacities in the lung parenchyma. Subsequent laboratory testing demonstrated a serum iodine level greater than 100,000 μg/L (normal: 40-92 μg/L), supporting the diagnosis of iododerma.

Intravenous methylprednisolone was initiated at 0.5 mg/kg/day and was tapered off over 5 weeks. Hydralazine was discontinued, and further iodinated contrast studies were avoided. The patient was successfully extubated, and his neurological examination improved. Repeat CT without contrast in 14 days showed a resolving nodule measuring 2.2 cm. Serum iodine levels normalized, and all skin manifestations resolved after several weeks.

## Discussion

Diagnosis of iododerma remains a challenge, as it may present with a variety of cutaneous morphologies that require careful consideration of the patient’s exposure history and clinical findings, which can include purpuric, acneiform, and vegetative lesions.^1^^,^^2^ Although iododerma lacks pathognomonic features, facial hemorrhagic vesiculopapules and bullae have been well described.^3^ In addition to the cutaneous features already reported in the literature, our patient developed retiform purpura, an under-recognized and newly reported manifestation of iododerma. The etiology underlying the morphologic changes of iododerma is not fully understood, although retiform purpura in this context may reflect vasculitic or vascular inflammatory processes associated with the condition.^1^

Iododerma is a diagnosis of exclusion and involves correlating the clinical presentation with elevated blood or urine iodine levels, supported by histopathologic changes that can often show haloed acellular structures resembling Cryptococcus, and an interstitial neutrophilic infiltrate, with occasional vasculitic changes.^1^^,^^2^^,^^4^^,^^5^ Nevertheless, histopathologic features of iododerma may vary depending on biopsy timing, further complicating the diagnosis in this clinically heterogeneous condition. Acute lesions show dense dermal neutrophilia, sometimes with suppurative folliculitis, whereas subacute lesions demonstrate pseudoepitheliomatous hyperplasia, neutrophilic microabscesses, and granulomatous inflammation, with possible necrosis and focal leukocytoclastic vasculitis.^6^

Iododerma may occur as a delayed hypersensitivity reaction within 1 hour to 1 week after iodine or contrast exposure.^7^ In individuals with normal renal function, iodine’s elimination half-life is approximately 90-120 minutes; however, underlying kidney disease increases the risk of iodine toxicity and subsequent iododerma.^6^ Mild cases are typically self-limited, whereas severe presentations, as in our patient, may require systemic corticosteroids.^7^ Iododerma most often involves the face, neck, and upper trunk, reflecting areas of high sebaceous gland concentration but may extend to the extremities in more disseminated or severe cases.^2^ Differential diagnosis includes cryptococcoid Sweet syndrome, cutaneous cryptococcosis, and AAV.

The clinical presentation of rapidly developing purpura that mimicked an AAV with atypical p-ANCA (antimyeloperoxidase/antiproteinase-3-negative) created a diagnostic challenge. Although the association between neutrophilic dermatoses and ANCA positivity has been reported, the importance is not fully elucidated in patients with iododerma, with only 3 reported cases demonstrating both a positive ANCA and a confirmed diagnosis of iododerma.^1^^,^^3^^,^^6^ Hydralazine-induced ANCA vasculitis accounting for the patient’s presentation was considered unlikely due to the histopathologic findings of cryptococcoid haloed structures supporting iododerma, and resolution coinciding with normalization of serum iodine levels. Notably, emerging reports indicate that iododerma can occur following intravenous iodinated contrast exposure in patients receiving hydralazine, as seen in our patient.^1^

Although iododerma is a rare cutaneous eruption of pathological iodine within the skin, there are deleterious sequelae associated with this dermatosis, including gastrointestinal bleeding, cardiac arrhythmias, and infiltration of internal organs, including the pulmonary system.^1^ A small number of cases in the literature have described an association between iododerma and lung nodule formation, as observed in our patient.^1^^,^^8^ Bronchial secretions are thought to increase in the setting of iodine toxicity and lead to nodular pulmonary infiltrates, which can be mistaken for lung cancer or disseminated fungal infection.^7^

This case highlights the development of retiform purpura secondary to iododerma in a patient with end-stage-renal disease, atypical p-ANCA positivity, hydralazine use, and exposure to intravenous iodinated contrast. As iododerma lacks pathognomonic features, its diagnosis should be considered in patients presenting with heterogeneous lesions and relevant risk factors, including potential pulmonary involvement. Iododerma should remain in the differential diagnosis of retiform purpura, particularly in those with identifiable sources of iodine exposure or predisposing conditions.

## Conflicts of interest

None disclosed.
